# The clinical relevance of sole loss of chromosome Y in myeloid neoplasms

**DOI:** 10.1038/s41408-026-01515-w

**Published:** 2026-05-09

**Authors:** Sandra Huber, Stephan Hutter, Manja Meggendorfer, Christian Pohlkamp, Torsten Haferlach, Isolde Summerer

**Affiliations:** MLL Munich Leukemia Laboratory, Max-Lebsche-Platz 31, 81377 Munich, Germany

**Keywords:** Cytogenetics, Chromosome abnormality, Leukaemia

**Dear Editor:**

Loss of chromosome Y (LOY) is one of the most common age-related somatic genomic alterations in men with up to 20% of healthy men over 80 years of age showing LOY by conventional karyotyping [1–4]. However, it is also a frequent cytogenetic abnormality in myelodysplastic neoplasms (MDS) [5, 6]. Ouseph et al. showed that the clinical significance of LOY depended on the proportion of cells affected, demonstrating that a threshold of ≥75% of metaphases with LOY was strongly associated with morphological features of MDS and represented a disease-associated aberration rather than incidental age-related mosaicism [6]. MDS with isolated LOY has further been ascribed to the ‘very good’ risk category of the revised International Prognostic Scoring System (IPSS-R) [7] demonstrating its association with superior survival rates compared to other karyotypes and reduced risk of leukemic transformation [5]. In addition, LOY is also observed in the context of clonal hematopoiesis [8, 9] including patients with premalignant cytopenia (clonal cytopenia of undetermined significance, CCUS) [10]. Recent research has revealed that LOY promotes an immunosuppressive tumor microenvironment indicating an association between LOY and immune dysfunction [11]. Thus, in clinical practice it may be difficult to distinguish whether LOY is a disease-associated alteration or an incidental aging-associated mosaicism in the respective patient, which makes it challenging to assess the role and the diagnostic relevance of sole LOY in patients with (suspected) myeloid neoplasm (MN).

Our study aimed to genetically and clinically characterize patients with (suspected) MN with sole LOY to assess the relevance of LOY in routine hematologic diagnostics.

The cohort comprised bone marrow samples of 1 986 male individuals sent to our laboratory between 2007 and 2024 with (suspected) MN (median age: 78 years [44–96]; Suppl. Table S1). All cases showed LOY as the sole cytogenetic alteration in chromosome banding analysis (CBA, ≥20 metaphases analyzed) and in fluorescence in situ hybridization (FISH, ≥100 interphase nuclei of uncultured cells analyzed) [12]. All cases were analyzed cytomorphologically. The mutational status of ≥24 genes associated with MN was analyzed by targeted next generation sequencing in 864/1 986 cases (Suppl. Methods). All cases were categorized based on the results of the cytomorphological analysis: group 0 (gr0): no hematologic neoplasm, group 1 (gr1): likely no MN, group 2 (gr2): MN possible, group 3 (gr3): diagnosis of MN. Final group assignments were based on the cytomorphologist’s case-specific assessment at diagnosis using WHO criteria and thresholds, integrating clinical context but not molecular findings (details see Suppl. Methods). All patients gave their written informed consent for genetic analyses and to the use of laboratory results and clinical data for research purposes according to the Declaration of Helsinki. The study was further approved by the laboratory’s institutional review board.

Within the 1 986 cases with sole LOY, the clone size of LOY in CBA strongly correlated with clone size in FISH (Pearson’s r = 0.81) but was significantly larger in CBA compared to FISH overall (median: 70% vs. 65%; median individual clone size difference: 7%) and comparing groups (Suppl. Table S1; Suppl. Figure S1). High proliferation activity of the LOY clone during cell culturing (defined as CBA clone size ≥20% larger than FISH) was observed in 19% of cases. In the following, only CBA was considered for LOY clone size definition, though patterns and statistical significance remained the same when FISH results were used. LOY clone size showed a significant but weak correlation with age (Pearson’s r = 0.17; p < 0.001; Suppl. Figure S2). Comparison of the clone size revealed a significant continuous increase from group 0 to group 3 (median: gr0: 55%, gr1: 60%, gr2: 65%, gr3: 85%; p < 0.001; Suppl. Table S1; Fig. 1), demonstrating a strong association of LOY clone size with disease state. Despite the high median LOY clone size of group 3 (85%) LOY clone sizes were significantly lower in MPN (MPN: 71%, MDS/MPN: 91%, MDS: 85%, AL/AML: 90%; p = 0.004 for MPN vs. others; Suppl. Figure S3). Overall, the median age was similar across groups except for group 0 showing a slightly lower median age (Suppl. Table S1).

**Fig. 1:**
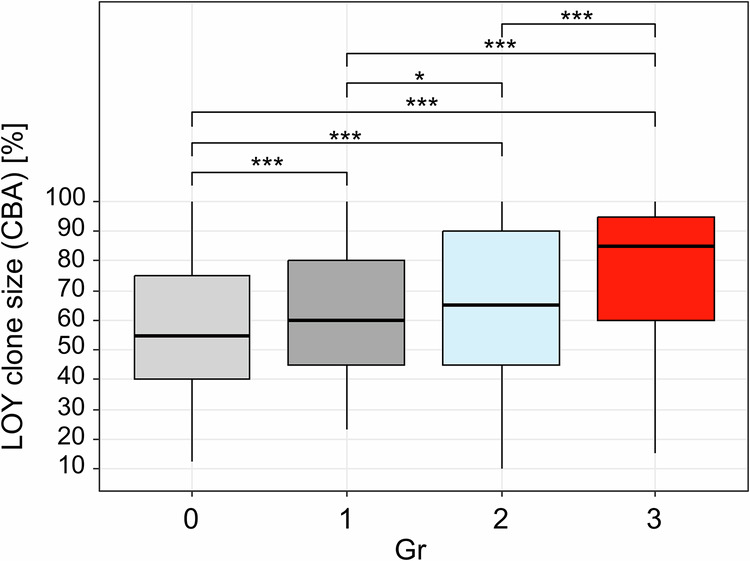
Differences in LOY clone size between four diagnostic groups. Mann-Whitney U test p-values: * p < 0.05, ** p < 0.01, *** p < 0.001; CBA: chromosome banding analysis; group (gr) 0: no hematologic neoplasm; gr1: likely no myeloid neoplasm (MN), gr2: MN possible, gr3: diagnosis of MN.

The proportion of cases carrying molecular mutations increased with disease state (gr0: 46%, gr1: 42%, gr2: 59%, gr3: 90%; Fig. 2A), as well as the number of genes mutated per case (median: gr0: 0 [0-4], gr1: 0 [0-3], gr2: 1 [0-5], gr3: 2 [0-8]; Suppl. Figure S4). LOY clone size was significantly larger in cases with mutations than in those without (85% vs. 55%; Fig. 2B). In order to contrast molecular patterns between large and small LOY clones we split the dataset at a cutoff point of 80% clone size in CBA (Suppl. Results; Suppl. Figure S5). LOY clones ≥80% showed significantly higher numbers of mutations compared to clones <80% (mean: 1.9 vs. 0.9; p < 0.001) comparable to previous studies [6, 13]. In our study, 83% of cases with LOY clones ≥80% carried at least one mutation, most commonly in *TET2* (45%), *SF3B1* (19%) and *ZRSR2* (14%), while only 54% of LOY clones <80% carried mutations, most commonly in *TET2* (18%), *DNMT3A* (11%) and *SF3B1* (10%) (Suppl. Figure S6A). Also the median variant allele frequency (VAF) of mutations was higher in cases with LOY clones ≥80% (34% vs. 10%, p < 0.001; Suppl. Figure S6B) in line with Ljungström et al. showing that men with higher levels of LOY tended to have higher VAF of mutations [8]. With respect to the mutation pattern within the different groups, *TET2* was the most frequently mutated gene in all four groups (gr0: 17%, gr1: 17%, gr2: 28%, gr3: 42%; Suppl. Figure S7). *TET2* frequencies in groups 0 and 1 were therefore comparable to cohorts displaying clonal hematopoiesis of indeterminate potential (CHIP) in the literature (reviewed in [14]). Besides that, the mutation profile was similar between group 0 and 1 but different from group 2 and group 3 (*DNMT3A*: more frequent in gr0/1; *SF3B1*: predominantly in gr3). With respect to clonal hierarchy, in the majority of cases the clone size of LOY (determined by FISH) was higher than or as high as co-occurring mutations (independent of diagnostic group, LOY clone size and mutated gene; Suppl. Figures S8/S9). Our data suggest that LOY together with molecular mutations indicates a high chance for the presence of MN and, that in this setting, LOY most likely is associated with MN and represents an early event in the pathogenesis.

**Fig. 2:**
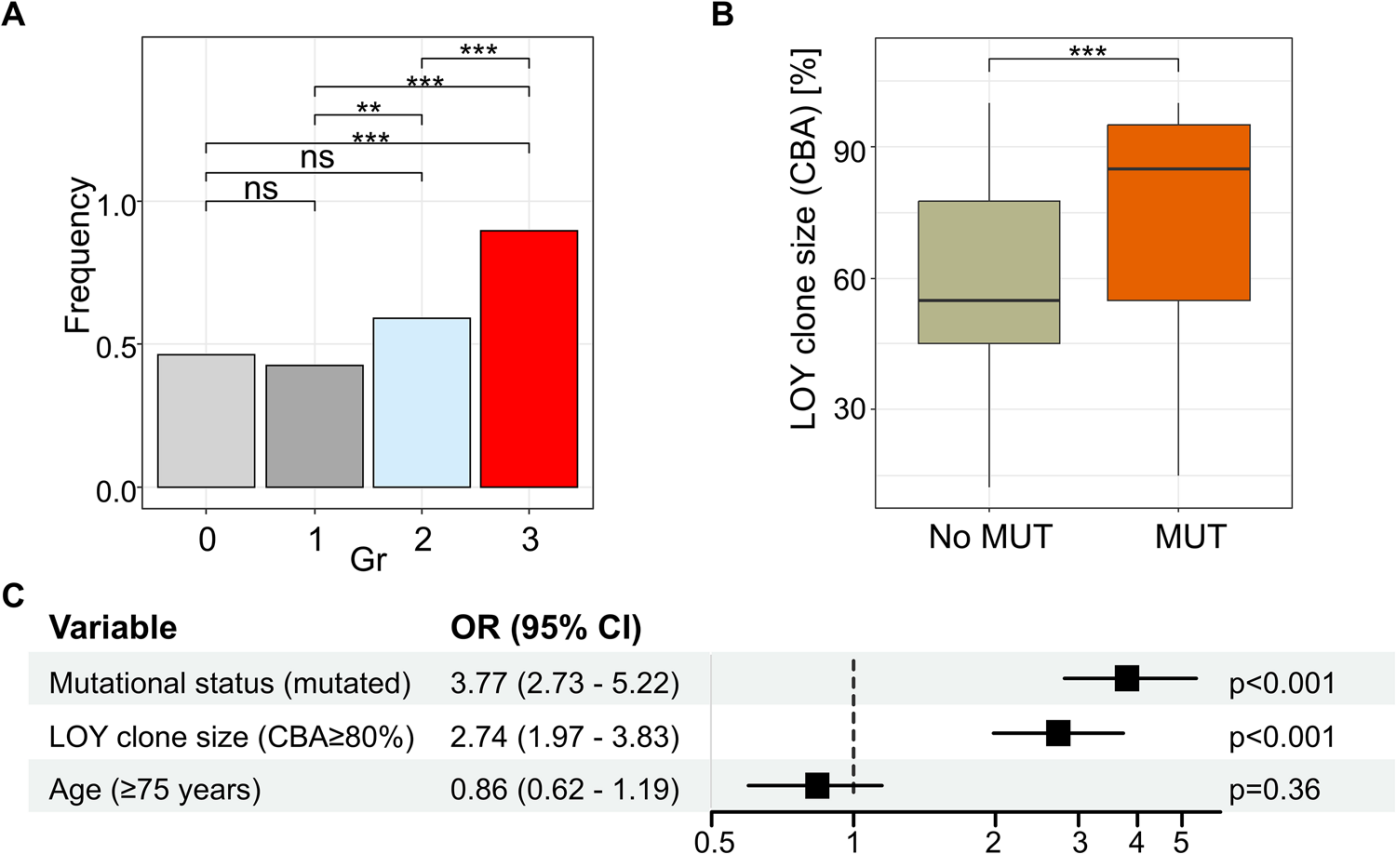
Mutational analysis in LOY cases. **A** Comparison of proportion of mutated cases between diagnostic groups using Fisher’s exact test. CBA: chromosome banding analysis; group (gr) 0: no hematologic neoplasm; gr1: likely no myeloid neoplasm (MN), gr2: MN possible, gr3: diagnosis of MN. **B** Comparison of CBA LOY clone size between non-mutated and mutated (MUT) cases using Mann-Whitney U test. Statistical test p-values: *p < 0.05, **p < 0.01, ***p < 0.001, ns: not significant. **C** Forest plot showing odds ratios (OR) of variables being associated with MN. CI confidence interval.

In order to facilitate assessing the relevance of LOY in routine diagnostics (especially when other methods like cytomorphology are lacking or unclear) we created a multivariate logistic regression model for cases with sole LOY. We modeled the probability of MN (i.e., being in gr2 or gr3) based on binary variables for age ( ≥ 75 years vs. <75 years), mutational status (mutated vs. wildtype) and CBA LOY clone size ( ≥ 80% vs. <80%). Mutational status and LOY clone size contributed significantly to the model (Fig. 2C). Mutational status had the largest effect in the model with an odds ratio of 3.77 for mutated cases, followed by LOY clone size with an odds ratio of 2.74 for clones ≥80%. Thus, not only the presence of mutations but also a large LOY clone is a predictor for MN independent of age. This implies that LOY is not only an age-associated phenomenon in these cases. This is further in line with Ouseph et al. showing that a high proportion of LOY is rather a disease-associated cytogenetic aberration than an incidental finding due to aging [6]. Another preclinical study by Zhang et al. further suggests LOY as a functional driver for clonal hematopoiesis and leukemogenesis [15]. Follow-up samples from cases without MN at initial diagnosis were available for 53 cases (median time span: 736 days) and were analyzed to assess the association of LOY clone size and the development of MN. Cases with LOY clones ≥80% (n = 15) showed a trend towards more frequent progression to MN than cases with LOY clones <80% (n = 38), however not reaching statistical significance due to small sample sizes (60% vs. 40%, not significant).

Limitations of our study are a lack of detailed follow-up in the majority of cases due to its retrospective design and the lack of a control group of healthy men with sole LOY without suspected hematological disease. As our study cohort exclusively consisted of men with suspected MN, our findings are restricted to a pre-selected high-risk population and cannot be applied to the general aging male population.

In summary, our results demonstrate a significant correlation between LOY clone size and the presence of MN in individuals with suspected MN. Thus, since mutations are an even stronger indicator of MN a molecular analysis should be performed in cases with a large LOY clone even if no definitive diagnosis of MN can be derived from cytomorphology. As individuals with large LOY clones, especially with co-occurring mutations, have a high probability of MN, close monitoring of these cases for a timely therapeutic intervention is indicated. Overall, although it remains challenging to evaluate the role of LOY in individual cases, LOY clone size and mutation status can help to assess the clinical relevance of LOY in routine diagnostics.

## Supplementary information

Supplemental Material

## Data Availability

The datasets generated during and/or analyzed during the current study are available from the corresponding author on reasonable request.
