# Supplementary material for: The clinical relevance of sole loss of chromosome Y in myeloid neoplasms
Source: Blood Cancer J. 2026 May 9;16(1):71. doi: 10.1038/s41408-026-01515-w (PMC13157478; doi:10.1038/s41408-026-01515-w)
Supplement: Supplementary file 1 — Supplemental Material [file 41408_2026_1515_MOESM1_ESM.pdf]

Supplementary Material

Supplementary Methods

Patients cohort and samples

All samples were sent to the MLL Munich Leukemia Laboratory between 05/2007 and 09/2024. Diagnoses from bone marrow were made based on cytomorphology, cytogenetics and molecular genetics as previously published (1-3).

Mutational analysis

Mutational data was retrieved from targeted next-generation sequencing (NGS; median coverage: 1 500x) available in 864 cases. In detail, we evaluated at least 24 genes associated with myeloid malignancies: *ASXL1, ASXL2, ATRX, BCOR, BCORL1, BRAF, CALR, CBL, CEBPA, CSF1R, CSNK1A1, CUX1, DDX41, DNMT3A, ETNK1, ETV6, EZH2, FBXW7, FLT3, GATA1, GATA2, GNB1, IDH1, IDH2, JAK2, KIT, KRAS, MPL, NF1, NOTCH1, NPM1, NRAS, PHF6, PIGA, PPM1D, PRPF8, PTPN11, RAD21, RUNX1, SETBP1, SF1, SF3A1, SF3B1, SH2B3, SMC1A, SMC3, SRSF2, STAG2, SUZ12, TET2, TP53, U2AF1, U2AF2, WT1, ZRSR2*. Protein-altering variants detected in myeloid genes were classified using a four-tier system based on ClinVar, Cosmic, functional impact predictors and gnomAD population frequency and only pathogenic or likely pathogenic mutations were considered (excluding variants of unknown significance and single nucleotide polymorphisms). A VAF detection limit of 3% was used for NGS. Variants with VAF below the sensitivity level were manually reviewed using the integrative genomics viewer (version 2.4.8 (4)), validated in a second sequencing run and considered as confirmed at a VAF of 1%. Structural variants/ fusions were analyzed by routine cytogenetics (encompassing chromosome banding analyses and FISH).

Clonal hierarchy

Clonal hierarchy of LOY compared to gene mutations was evaluated by comparing the proportion of LOY carrying cells from FISH analysis (interphase nuclei of uncultured cells) to the VAF of mutations. To make these directly comparable, FISH values and VAFs of X-linked genes were halved. Furthermore, VAFs of mutations in autosomal genes with values >50% were also halved, as this indicates an overlap with regions of copy-neutral loss of heterozygosity or mono-allelic gene deletions. Adjusted values within 10% of each other were considered to be residing within the same clone.

Cytomorphological group assignment

The final diagnoses were based on the individual and case-specific assessment performed by the cytomorphologist at the time of initial diagnosis. In this context, not only the degree of dysplasia was considered, but also clinical background information such as medication use, other toxic or reactive influences on the bone marrow, patient's age, degree of cytopenia and comorbidities. With the focus on group 1 and 2, group 1 included cases where a malignant condition was possible, but less likely than a non-malignant condition and group 2 cases where a malignant condition is likely, but a non-malignant condition is possible. In summary, the diagnostic judgment reflected the relative weighting between the extent of dysplasia and the plausibility of alternative, non-MDS-related causes of dysplastic changes. WHO criteria including corresponding thresholds in assessment of dysplasia and cytopenia were applied, molecular findings did not influence final diagnoses.

Statistical analysis

For statistical analyses R version 4.2.2 (R Foundation for Statistical Computing, Vienna, Austria) and SPSS version 19.0 (IBM Corporation, Armonk, NY) were used.

Dichotomous variables were compared using Fishers exact or chi-square test. For comparison of median values, the two-sided Mann-Whitney U test was applied. All results were considered significant at $p<0.05$.

In order to identify an optimal cutoff for separating our cohort into two groups carrying large vs. small LOY clones a receiver operating characteristic (ROC) analysis on LOY clone size from CBA as a predictor for the presence of a myeloid neoplasm (MN, gr0 + gr1 vs. gr2 + gr3) was performed. The optimal cutoff was determined by maximization of Youden's index (5), which combines specificity and sensitivity.

Supplementary Results

LOY clone size ROC analysis

ROC analysis on CBA LOY clone size as a predictor of MN revealed an area under the curve of 0.67 (Suppl. Figure S5A). A clone size threshold of 79.5% was found to be the optimal cutoff (Suppl. Figure S5B). For the sake of simplicity, we chose 80% as a cutoff for further analysis, which divided the dataset into groups of 829 (≥80%) and 1 157 (<80%) cases.

Supplementary Tables and Figures

Table S1. Cohort overview

Characteristics	All cases	Gr0	Gr1	Gr2	Gr3
Cases (n)	1 986	369	345	499	773*
Age (years; median [range])	78 [44-96]	76 [52-91]	79 [49-96]	79 [49-94]	78 [44-95]
LOY clone size (CBA, median)	70%	55%	60%	65%	85%
LOY clone size (FISH, median)	65%	44%	53%	60%	80%

CBA: chromosome banding analysis; FISH: fluorescence in situ hybridization; * MDS: n=458, MDS/AML border cases: n=9, AML or other acute leukemia: n=92, MDS/MPN: n=129, MPN: n=81, SM-AHN (systemic mastocytosis with associated hematological neoplasm): n=4.

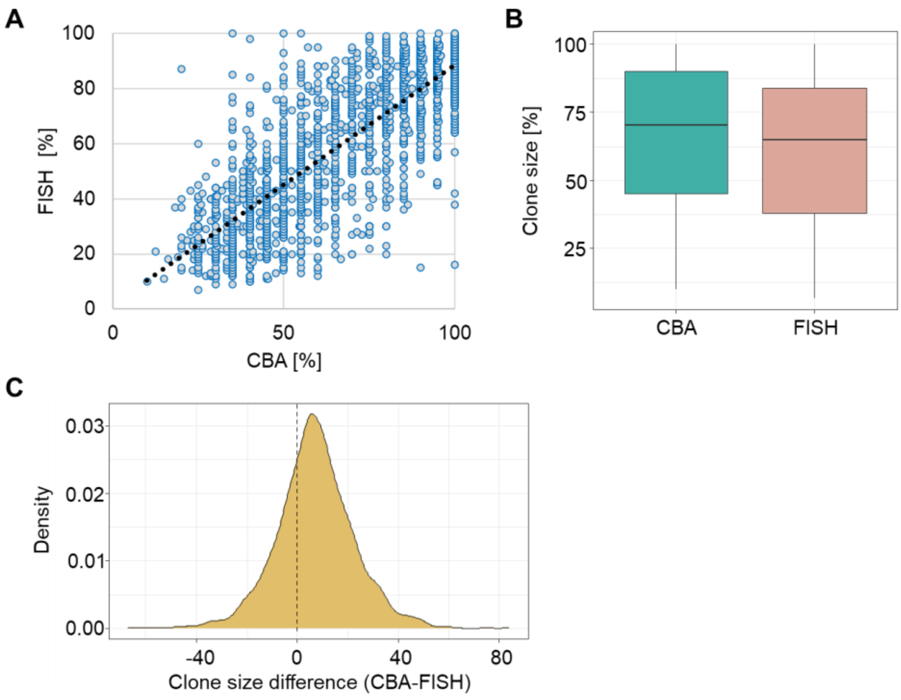

Supplementary Figure S1: LOY clone size CBA vs. FISH. (A) Correlation between LOY clone size in CBA and FISH (Pearson's r=0.81). **(B)** Boxplot showing LOY clone sizes of CBA and FISH. **(C)** Density plot illustrating the LOY clone size difference between CBA and FISH.

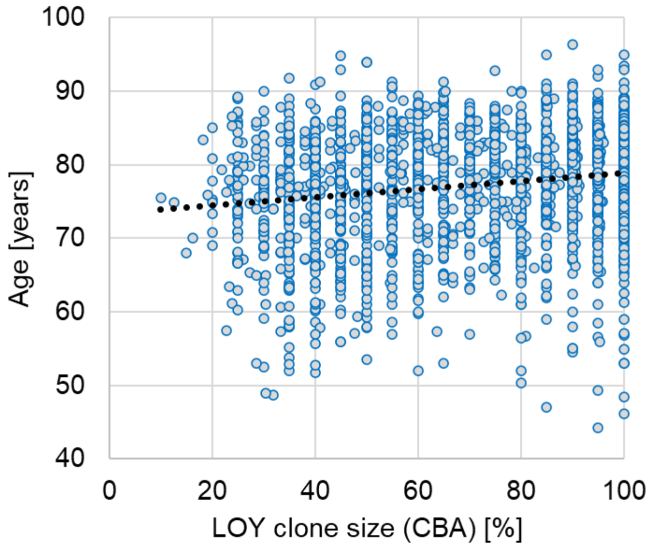

Supplementary Figure S2: Correlation between LOY clone size in CBA and age. Pearson's r=0.17.

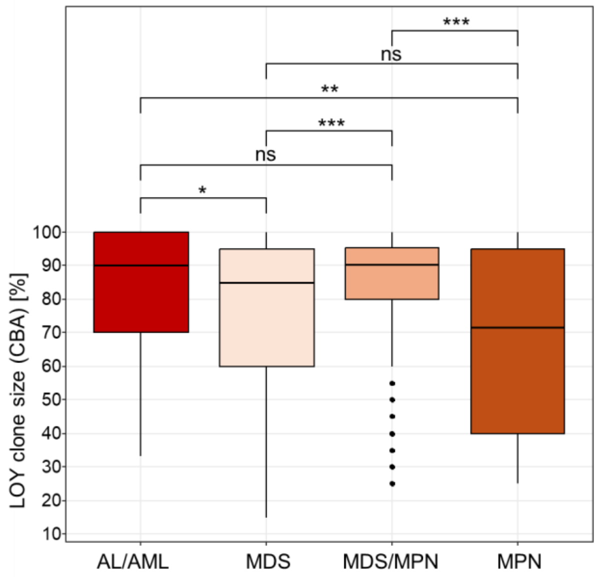

Supplementary Figure S3: LOY clone sizes in group 3. Boxplot of LOY clone sizes in CBA for the different MN sub-groups in group 3 (diagnosis of myeloid neoplasm/MN). Only sub-groups with more than 10 cases are shown. Mann-Whitney U test p-values: * p<0.05, ** p<0.01, *** p<0.001, ns: not significant.

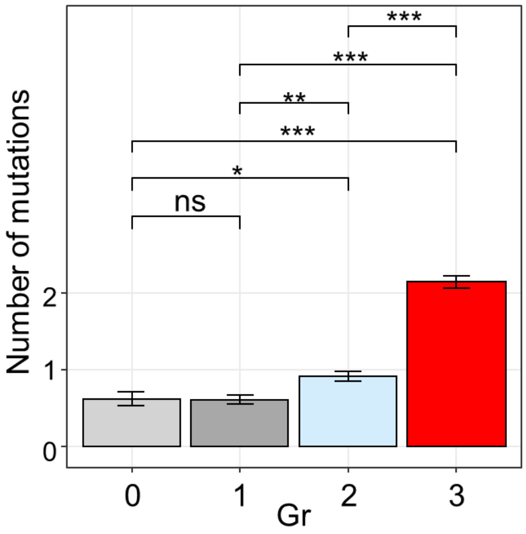

Supplementary Figure S4: Comparison of mean number of mutated genes per case between diagnostic groups. Mann-Whitney U test p-values:* p<0.05, ** p<0.01, *** p<0.001, ns: not significant. group (gr) 0: no hematologic neoplasm; gr1: likely no myeloid neoplasm (MN), gr2: MN possible, gr3: diagnosis of MN.

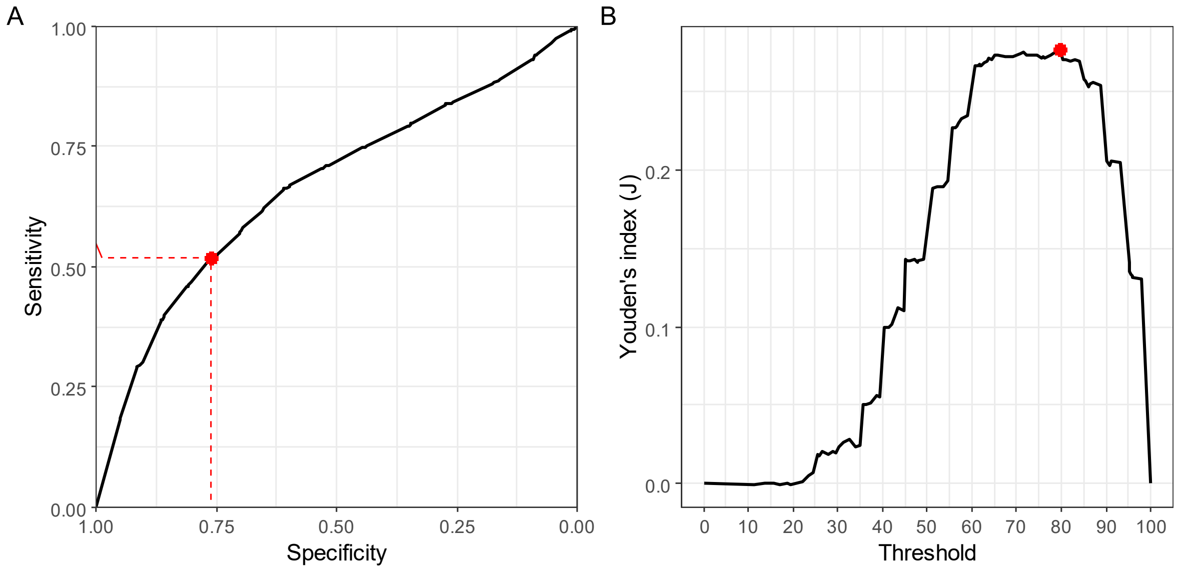

Supplementary Figure S5: ROC analysis of LOY CBA clone size predicting the presence of MN. (A) ROC curve, **(B)** Youden's index (J) for different LOY clone size thresholds. The location of maximized J is shown in red.

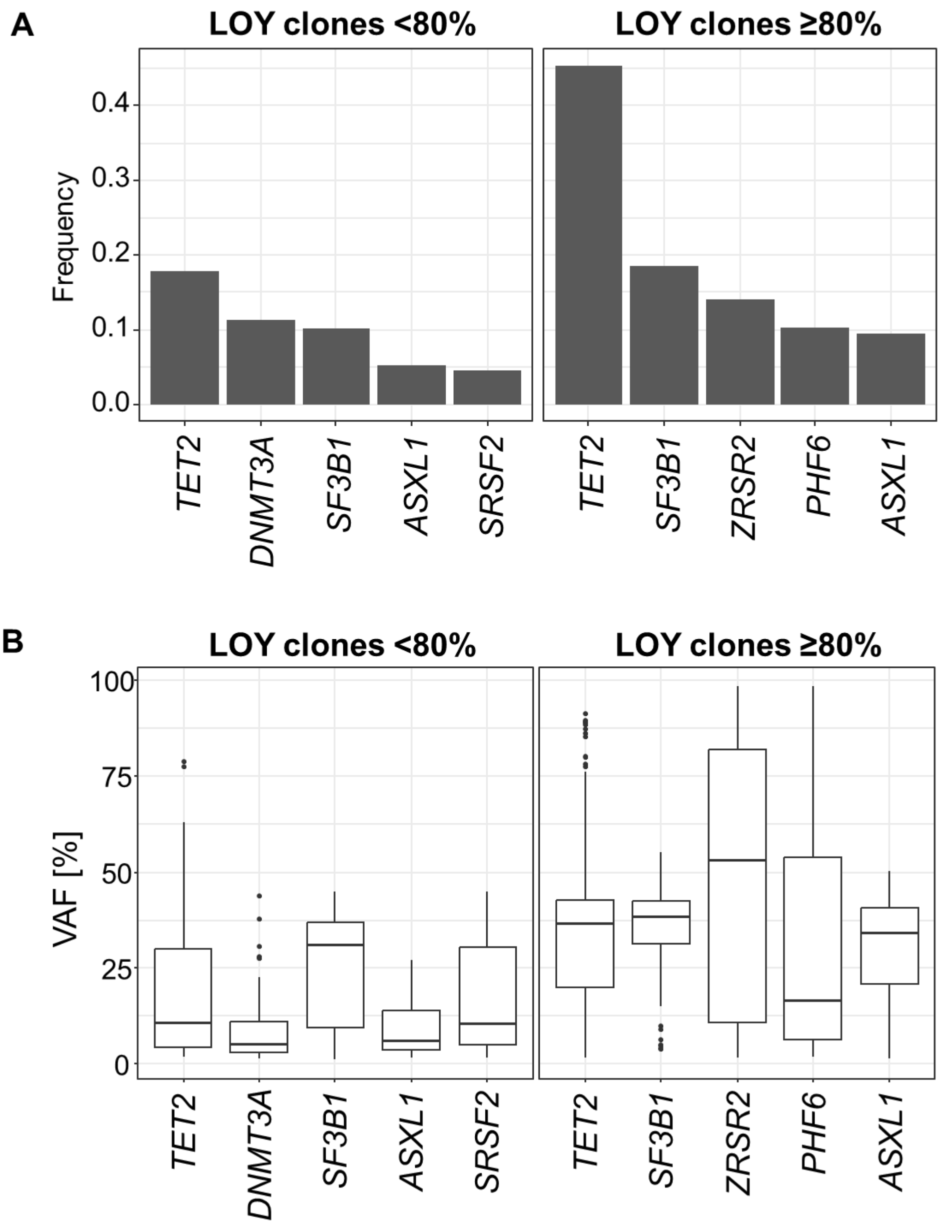

Supplementary Figure S6: (A) Proportion of mutated cases for the five most frequently mutated genes within cases with CBA LOY clone size <80% (n=465) and ≥80% (n=399). **(B)** Variant allele frequencies (VAF) of the five most frequently mutated genes within cases with CBA LOY clone size <80% and ≥80%. Boxplots with median VAF are shown.

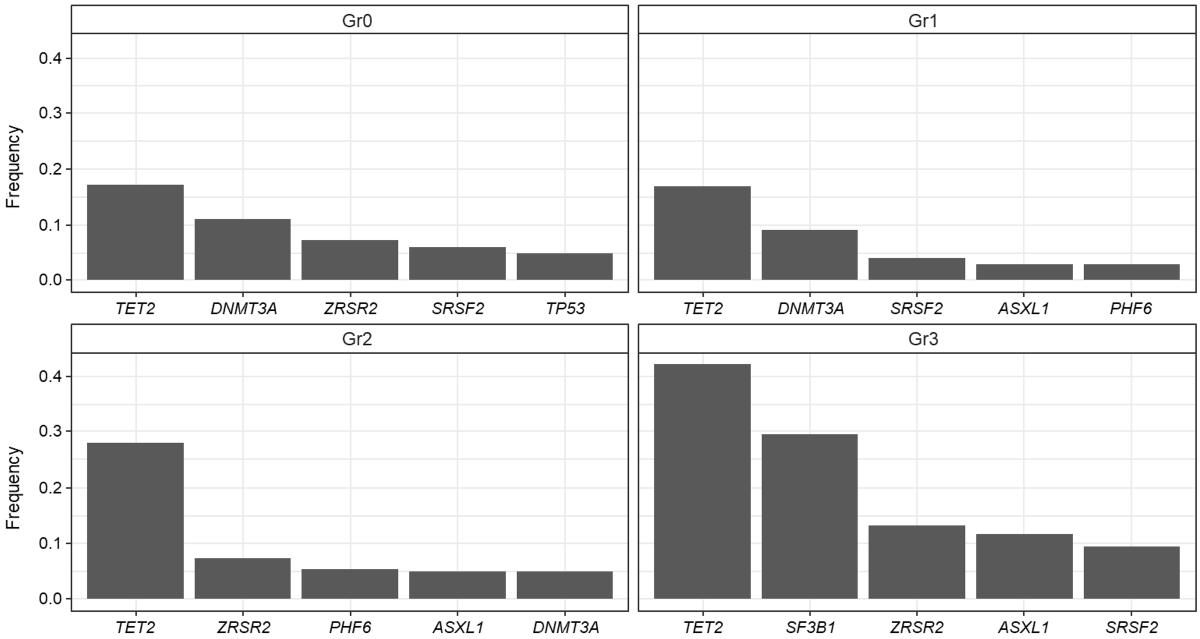

Supplementary Figure S7: Proportion of mutated cases for the five most frequently mutated genes within the different diagnostic groups; group (gr) 0: no hematologic neoplasm; gr1: likely no myeloid neoplasm (MN), gr2: MN possible, gr3: diagnosis of MN.

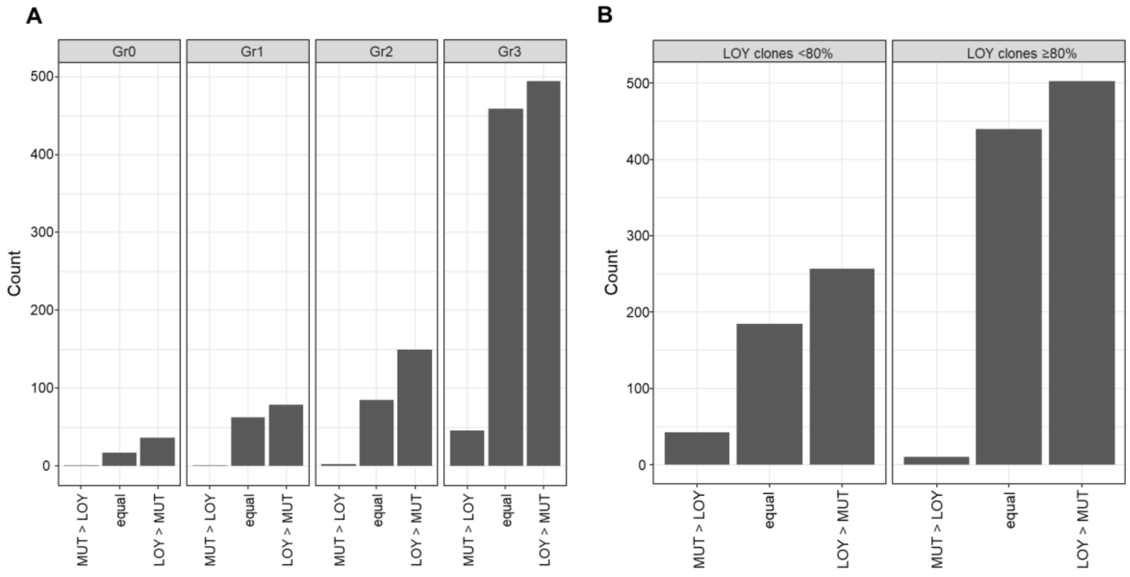

Supplementary Figure S8: Clonal hierarchy analysis. (A) Clonal hierarchy of LOY compared to co-occurring mutations (MUT) within the different diagnostic groups. Group (gr) 0: no hematologic neoplasm; gr1: likely no myeloid neoplasm (MN), gr2: MN possible, gr3: diagnosis of MN. **(B)** Clonal hierarchy of LOY compared to co-occurring mutations (MUT) within cases with CBA LOY clone size <80% and ≥80%.

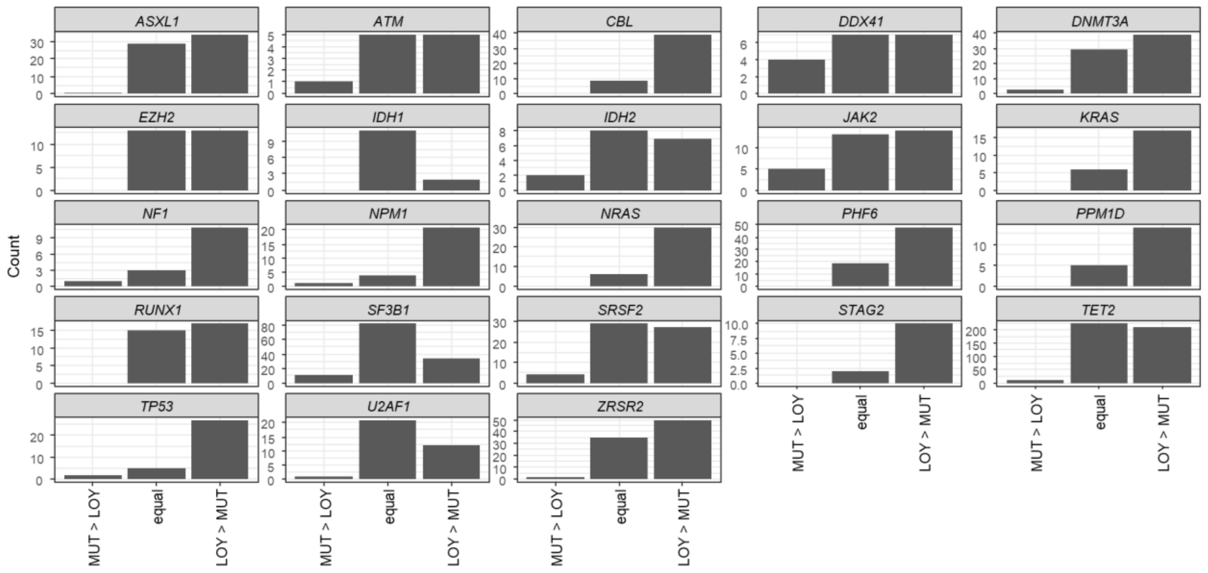

Supplementary Figure S9: Clonal hierarchy analysis of LOY compared to co-occurring mutations (MUT) for the different genes. Only genes mutated ≥10 times are shown.

References

1. Schoch C, Schnittger S, Bursch S, Gerstner D, Hochhaus A, Berger U, et al. Comparison of chromosome banding analysis, interphase- and hypermetaphase-FISH, qualitative and quantitative PCR for diagnosis and for follow-up in chronic myeloid leukemia: a study on 350 cases. Leukemia. 2002;16(1):53-9.
2. Haferlach T, Kern W, Schoch C, Hiddemann W, Sauerland MC. Morphologic dysplasia in acute myeloid leukemia: importance of granulocytic dysplasia. J Clin Oncol. 2003;21(15):3004-5.
3. Kern W, Voskova D, Schoch C, Hiddemann W, Schnittger S, Haferlach T. Determination of relapse risk based on assessment of minimal residual disease during complete remission by multiparameter flow cytometry in unselected patients with acute myeloid leukemia. Blood. 2004;104(10):3078-85.
4. Robinson JT, Thorvaldsdóttir H, Winckler W, Guttman M, Lander ES, Getz G, Mesirov JP. Integrative genomics viewer. Nat Biotechnol. 2011;29(1):24-6.
5. Youden WJ. Index for rating diagnostic tests. Cancer. 1950;3(1):32-5.
